# Clinical application of a lung cancer organoid (tumoroid) culture system

**DOI:** 10.1038/s41698-021-00166-3

**Published:** 2021-04-12

**Authors:** Etsuko Yokota, Miki Iwai, Takuro Yukawa, Masakazu Yoshida, Yoshio Naomoto, Minoru Haisa, Yasumasa Monobe, Nagio Takigawa, Minzhe Guo, Yutaka Maeda, Takuya Fukazawa, Tomoki Yamatsuji

**Affiliations:** 1 Department of General Surgery, Kawasaki Medical School, Okayama, Japan; 2 General Medical Center Research Unit, Kawasaki Medical School, Okayama, Japan; 3 Department of Thoracic Surgery, Kurashiki Central Hospital, Kurashiki, Japan; 4 Professor with Special Assignment, Kawasaki Medical School, Okayama, Japan; 5 Department of Pathology, Kawasaki Medical School, Okayama, Japan; 6 Department of General Internal Medicine 4, Kawasaki Medical School, Okayama, Japan; 7 Perinatal Institute, Division of Neonatology, Perinatal and Pulmonary Biology, Cincinnati Children’s Hospital Medical Center (CCHMC) and Department of Pediatrics, The University of Cincinnati College of Medicine (UC-COM), Cincinnati, OH USA

**Keywords:** Lung cancer, Mesothelioma

## Abstract

Despite high expectations for lung tumoroids, they have not been applied in the clinic due to the difficulty of their long-term culture. Here, however, using AO (airway organoid) media developed by the Clevers laboratory, we succeeded in generating 3 lung tumoroid lines for long-term culture (>13 months) from 41 lung cancer cases (primary or metastatic). Use of nutlin-3a was key to selecting lung tumoroids that harbor mutant p53 in order to eliminate normal lung epithelial organoids. Next-generation sequencing (NGS) analysis indicated that each lung tumoroid carried *BRAF*^*G469A*^, *TPM3-ROS1* or *EGFR*^*L858R*^*/RB1*^E737*^, respectively. Targeted therapies using small molecule drugs (trametinib/erlotinib for *BRAF*^*G469A*^, crizotinib/entrectinib for *TPM3-ROS1* and ABT-263/YM-155 for *EGFR*^*L858R*^*/RB1*^E737*^) significantly suppressed the growth of each lung tumoroid line. AO media was superior to 3 different media developed by other laboratories. Our experience indicates that long-term lung tumoroid culture is feasible, allowing us to identify NGS-based therapeutic targets and determine the responsiveness to corresponding small molecule drugs.

## Introduction

The treatment of lung cancer has been dramatically improved in the past 17 years with the discovery of driver oncogenes and the development of molecularly targeted drugs that bind them, including osimertinib for mutant *EGFR* or crizotinib for *ROS1* fusions^1,2^. However, there are driver oncogenes for which molecularly targeted drugs have not yet been identified. For example, patients with *BRAF*^*V600E*^, which is seen in melanoma, colorectal cancer and non-small cell lung cancer (NSCLC), are currently treated using vemurafenib in the clinic; however, there are no molecularly targeted drugs to treat patients with *BRAF*^*G469A*^, which is occasionally seen in NSCLC^3^. Importantly, such cancers treated with small molecule drugs, including osimertinib, crizotinib and vemurafenib, always recur by acquiring different drug-resistance mechanisms. The generation of an avatar cancer cell line derived from each patient’s cancer will meet the need for personalized medicine to identify small molecule drugs for each patient so as to eradicate lung cancer cells that harbor previously untargeted driver oncogenes and/or drug-resistance pathways.

To date, >300 lung cancer cell lines have been generated in vitro by a two-dimensional (2D) culture system^4^ but the generation rate is low at <5%^5^, which is not practical for personalized medicine. Recently, in vivo patient-derived xenografts (PDXs) using mice have been applied for the same purpose. The generation rate is as high as 30%; however its use for personalized medicine is limited because of its cost, which is much higher than the in vitro 2D culture system^6^.

In 2009, Clevers and colleagues developed a method to culture normal intestinal cells as organotypic structures in an in vitro three-dimensional (3D) culture system by embedding the cells in Matrigel (basement membrane extracellular matrix)^7^. The culture media consists of a ROCK inhibitor (anoikis/apoptosis inhibitor) and growth factors (R-spondin 1, EGF and Noggin). Notably, this culture system has been applied to culture human colon cancer cells as organotypic structures as well, the success rate of which is as high as >80%^8^ (hereafter, organotypic structures from normal cells are referred to as organoids, and organotypic structures from cancer cells are referred to as tumoroids^9,10^). The cost to maintain tumoroids is comparable to the 2D culture system. Since tumoroids are applicable for long-term culture (>6 months), which allows for genetic and drug sensitivity tests in a reproducible manner, this 3D culture system has been clinically used for personalized medicine^11^.

In 2013, Inoue and colleagues reported that they succeeded in culturing lung cancer cells as 3D tumoroids embedded in Matrigel using human embryonic stem cell culture media in addition to fibroblast growth factor 2 (FGF2)^12^. The reported success rate was 80%; however, they did not report genetic alterations in DNA extracted from the tumoroids and did not document whether their system was suitable for long-term culture.

In 2019, Clevers and colleagues reported a 3D tumoroid system to culture lung cancer cells. The major difference from their colon tumoroid culture system reported in 2009 was that key growth factors (FGF7 and FGF10) that are expressed in normal lung were added to the colon tumoroid media, which they called AO (airway organoid) media^9^. They used nutlin-3a (MDM-2 inhibitor^13^), which activates wild-type p53, to eliminate normal lung epithelial cells, thereby succeeding in culturing pure lung tumoroids that harbor mutant p53 (Supplementary Fig. 1a, b). Their success rate was 28%. Importantly, in their study, they documented genetic alterations from DNA extracted from the generated tumoroids that matched to those of the parental tumor.

In the same year, Jang and colleagues reported that they succeeded in culturing long-term lung tumoroids (>10 passages) at a success rate of 70% using media containing FGF2 and EGF but no other growth factors. Importantly, they also documented genetic alterations from DNA extracted from their lung tumoroids^14^. Tsao and colleagues^15^ also reported a long-term 3D lung tumoroid system in which they used media containing EGF, Noggin, FGF4, FGF10 and smoothened agonist (SAG); however, their success rate was 15% lower than the culture systems reported by Inoue (80%) and Jang (70%).

Currently, the discrepancy in lung tumoroid generation rates (%) for long-term culture is a controversial issue. Notably, in 2018, Swanton and colleagues reported that, when using fibroblast conditioned media with EGF, the generation rate of the long-term lung tumoroids was 8% due to the overgrowth of normal lung epithelial cells compared to that of lung cancer cells^16^. In 2020, Voest and colleagues^17^, also using the AO media described by Clevers and colleagues, reported that their lung tumoroid generation rate was 17%, due to the same reason that normal lung epithelial cells grow faster than lung cancer cells. The difficulty in culturing lung tumoroids over normal lung epithelial organoids might be implicated by the fact that 28 colon tumoroids have been deposited to ATCC (American Type Culture Collection) while only 2 lung tumoroids have been deposited to ATCC (Supplementary Table 1). Of note, 37 colon tumoroids have been genetically analyzed by the Human Cancer Models Initiative (HCMI) associated with ATCC (https://cellmodelpassports.sanger.ac.uk) but no lung tumoroids as of December 2020.

In the present study, we set out to determine whether we could implement a lung tumoroid culture system using lung cancer specimens obtained from our hospital, hoping to use the lung tumoroids for personalized medicine. For implementation, we started using the AO media system reported by Clevers and colleagues^9^ and assessed the long-term generation rate. Using the generated long-term tumoroid lines, we extracted DNA and RNA and analyzed their genetic alterations with next-generation sequencing (NGS), including exome-seq and RNA-seq. Based on the genetic alterations obtained by the analysis, we treated the lung tumoroids with small molecule drugs that were targeted to the genetic alterations. In addition to the system using AO media, we further tested three different 3D tumoroid culture systems reported by other researchers, including Inoue, Jang, Tsao, and colleagues^12,14,15^, to determine which tumoroid culture system is most efficient.

## Results

### Summary of patient-derived lung tumoroids generated in our hospital

Using the lung tumoroid/organoid culture system that contains AO media developed by Clevers and colleagues^9^, we set out to generate lung tumoroids derived from primary lung tumors, lymph node metastases and/or malignant pleural effusions from lung cancer patients in our hospital (Table 1). We also set out to generate organoids from adjacent normal lung tissue to compare with lung tumoroids. Tumoroids/organoids formations were observed in 34 of 41 cases. As shown in Table 1, *EGFR* mutations were detected in genomic DNA obtained from either tumor tissues or malignant pleural effusions from eleven lung cancer patients using genetic tests by a certified laboratory. In order to confirm whether the generated tumoroids harbor the same *EGFR* mutations of the parental tumors, Sanger sequencing was performed using genomic DNA obtained from the tumoroids (Supplementary Fig. 2a) and from the corresponding parental tumors. Whereas *EGFR* mutations were detected in the genomic DNA from the parental tumors, which were consistent with the results obtained by the certified laboratory, such *EGFR* mutations were not detected in the DNA extracted from the generated tumoroids (Supplementary Fig. 2b). In addition to this genetic inconsistency between the generated tumoroids and the parental tumor tissues, immunohistochemistry (IHC) analysis indicated that the expression pattern of lung cancer biomarkers (e.g., NKX2-1/TTF-1) in the generated tumoroids were not consistent with that of the parental tumor tissues (Supplementary Fig. 2c). These results indicate that the tumoroids generated from the lung tumor tissues were not in fact tumoroids but normal organoids originating from normal lung epithelial cells that also exist in lung tumor tissues, the results of which are consistent with those reported by Swanton, Voest, and colleagues^16,17^.

**Table 1:** List of lung cancer samples used for this study.

Case	Sample number	Sex	Age	Lung cancer type	Subtype	Stage	Sample type	Organoid formation	Tumoroid formation	Driver oncogene	Passage number
1	L1	M	80	Small cell carcinoma		pStage IA3	Surgical specimen	◯	X		N/A
2	L2	M	76	Adenocarcinoma		pStage IIIA	Surgical specimen	◯	X		N/A
3	L3	M	79	Adenocarcinoma	Acinar	pStage IA2	Surgical specimen	◯	X		N/A
4	L4	F	56	Adenocarcinoma	Acinar	pStage IVA	Surgical specimen	◯	X	EGFR (Exon 18 G719S)	N/A
5	L5	M	73	Adenocarcinoma	Solid	pStage IA2	Surgical specimen	◯	◎		More than 22 months, passage >92
6	L6	F	77	Adenocarcinoma	Mucinous	pStage IB	Surgical specimen	◯	X		N/A
7	L7	M	76	Squamous cell carcinoma	Poor	pStage IIB	Surgical specimen	◯	X		N/A
8	L8	F	75	Adenocarcinoma		pStage IA3	Surgical specimen	◯	X		N/A
9	L9	M	79	Adenocarcinoma	Papillary	pStage IA2	Surgical specimen	◯	X		N/A
10	L10	F	82	Adenocarcinoma		cStage IVA	Pleural effusion	X	X		N/A
11	L11	M	75	Adenocarcinoma	Papillary	pStage IIA	Surgical specimen	◯	X	EGFR (Exon 21 L858R)	N/A
12	L12	M	69	Adenocarcinoma	Lepidic	pStage IA2	Surgical specimen	◯	X	EGFR (Exon 21 L858R)	N/A
13	L13	F	70	Adenocarcinoma		pStage IIA	Surgical specimen	◯	X	EGFR (Exon 21 L858R)	N/A
14	L14	M	83	Adenocarcinoma	Solid	pStage IB	Surgical specimen	◯	X		N/A
15	L15	M	62	Adenocarcinoma		pStage IA1	Surgical specimen	◯	X	EGFR (Exon 18 G719A)	N/A
16	L16	M	77	Squamous cell carcinoma	Moderate	pStage IA2	Surgical specimen	◯	X		N/A
17	L17	M	69	Adenocarcinoma	Lepidic	pStage IA2	Surgical specimen	◯	X	EGFR (Exon 21 L858R)	N/A
18	L18	M	75	Adenocarcinoma	Solid	pStage IA3	Surgical specimen	◯	X	EGFR (Exon 19 del)	N/A
19	L19	F	65	Adenocarcinoma	Acinar	pStage IIIA	Surgical specimen	◯	◎		More than 22 months, passage >86
20	L20	M	81	Squamous cell carcinoma		pStage IB	Surgical specimen	◯	X		N/A
21	L21	F	70	Adenocarcinoma	Mucinous	pStage IA2	Surgical specimen	◯	X		N/A
22	L23	M	77	Adenocarcinoma		pStage IA2	Surgical specimen	◯	X		N/A
23	L24	M	69	Adenocarcinoma	Acinar	pStage IA2	Surgical specimen	◯	X	EGFR (Exon 18 G719A, S720Y)	N/A
24	L25	F	56	Adenocarcinoma in situ		pStage 0	Surgical specimen	◯	X	EGFR (Exon 18 G719S)	N/A
25	L26	F	70	Adenocarcinoma	Lepidic	pStage IA1	Surgical specimen	◯	X		N/A
26	L27	M	58	Adenocarcinoma	Mucinous	pStage IIIA	Surgical specimen	◯	X		N/A
27	L28	M	52	Adenocarcinoma	Acinar	pStage IIIA	Surgical specimen	◯	X		N/A
28	L29	F	74	Pleomorphic carcinoma		pStage IB	Surgical specimen	◯	X		N/A
29	L30	M	71	Squamous cell carcinoma	Moderate	pStage IIA	Surgical specimen	◯	X		N/A
30	L31	M	84	Adenocarcinoma	Papillary	pStage IA1	Surgical specimen	X	X		N/A
31	L32	F	72	Adenocarcinoma	Lepidic	pStage IA1	Surgical specimen	◯	X		N/A
32	L33	F	50	Adenocarcinoma	Lepidic	pStage IA2	Surgical specimen	◯	X		N/A
33	L34	M	77	Squamous cell carcinoma		pStage IIIA	Surgical specimen	◯	X		N/A
34	L35	M	70	Small cell carcinoma		pStage IB	Surgical specimen	X	X		N/A
35	L37	M	86	Pleomorphic carcinoma		pStage IIB	Surgical specimen	X	X	EGFR (Exon 19 del)	N/A
36	L38	F	71	Adenocarcinoma	Lepidic	pStage IA1	Surgical specimen	X	X		N/A
37	L39	F	77	Squamous cell carcinoma		pStage IIA	Surgical specimen	◯	X		N/A
38	L40	F	86	Adenocarcinoma		pStage IIIA	Surgical specimen	X	X		N/A
39	L41	M	70	Adenocarcinoma		T2aNxMx	Surgical specimen	X	X		N/A
40	L42	M	77	Squamous cell carcinoma		pStage IIA	Surgical specimen	◯	X		N/A
41	L43	F	73	Adenocarcinoma		pStage IVB	Pleural effusion	◯	◎	EGFR (Exon 20 T790M loss, Exon 21 L858R)	>13 months, passage >36

In order to eliminate such normal lung organoids, Clevers and colleagues used nutlin-3a (MDM-2 inhibitor), which activates p53 in normal lung epithelial cells and induces apoptosis, thereby eliminating such cells while lung tumor cells that harbor mutant p53 survive^13^ (Supplementary Fig. 1a, b). Using this strategy that includes nutlin-3a in the AO media, we were also able to generate lung tumoroids. To validate that these lung tumoroids indeed originated from lung tumor cells but not normal lung epithelial cells, we used karyotyping of chromosomes obtained from the lung tumoroids, which was previously used by Garnett and colleagues to validate human esophageal tumoroids^18^. In the end, out of the 41 lung cancer cases in our hospital, we succeeded in generating genetically validated lung tumoroids each from a primary lung tumor (Patient-Derived Tumoroid-Lung Adenocarcinoma; PDT-LUAD#5), from a lymph node metastasis (PDT-LUAD#19) and from a malignant pleural effusion (PDT-LUAD#43) (7% success rate), details of which are described below. Notably, we were able to culture these three lung tumoroid lines over 36 passages (>13 months, Table 1), thus they may be deposited in public biobanks (e.g., ATCC) upon request.

### Generation of tumoroids derived from early stage primary lung adenocarcinoma

**Fig. 1:**
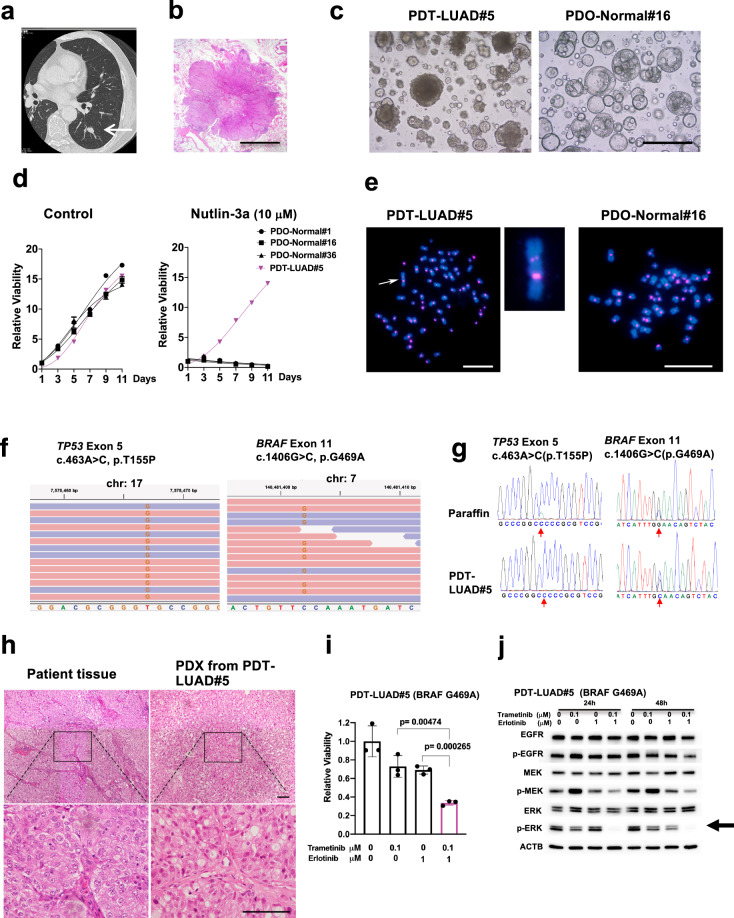
Patient-derived tumoroids (PDTs) from early stage primary lung cancer with a *BRAF* mutation (PDT-LUAD#5). **a** The nodule located in the left lower lobe was found by a CT scan (white arrow). **b** Lung cancer with lobulated edges is shown in Loupe view by hematoxylin-eosin (HE) staining. Scale bar, 5 mm. **c** Shown are bright-field microscopy images of PDT-LUAD#5 and PDO-Normal#16. Scale bar, 500 μm. **d** Cell viability assays on tumoroids or organoids were conducted over 11 days with or without nutlin-3a (10 μM). Lung tumoroids (PDTs) and normal lung organoids (PDOs) were seeded into 96-well white polystyrene plates at a density of 1 × 10^3^ cells with 4 μl of Cultrex growth factor reduced BME type 2 (Matrigel) per well 24 h before treatment. Cell viability assay was performed as described in the Methods section. **e** Shown are metaphase FISH images of PDT-LUAD#5 and PDO-Normal#16. An aberrant chromosome indicated by a white arrow was seen in PDT-LUAD#5. No chromosome abnormalities are seen in PDO-Normal#16. Scale bars, 20 μm. **f** Exome-seq detected a biallelic *TP53* c.463A > C (p.T155P) mutation and monoallelic *BRAF* c.1406G > C (p.G469A) mutation from PDT-LUAD#5. Red and blue lines represent forward and reverse reads, respectively. **g** Sanger sequencing confirmed the *TP53* and *BRAF* mutations seen in **f** in DNA from both paraffin-embedded parental tissue and lung tumoroids (PDT-LUAD#5). **h** Shown are HE stained images of the parental lung cancer tissue (left) and the PDX (patient-derived xenografts) derived from PDT-LUAD#5 (right) (magnified in lower image). Scale bar, 100 μm. **i** Combination treatment of trametinib and erlotinib significantly suppressed the viability of PDT-LUAD#5 72 h after treatment. Cell viability assay was performed as described in **d**. Data are shown as mean ± SD. **j** The combination treatment of trametinib and erlotinib suppressed phosphorylation of ERK (arrow) both 24 and 48 h after treatment.

A 73-year-old man was diagnosed with suspected lung cancer using a computer tomography (CT) scan (Fig. 1a). A left lower lobectomy and mediastinal lymphadenectomy were performed to resect the lung tumor via video-assisted thoracic surgery. Postsurgical pathological diagnosis indicated that the tumor was invasive adenocarcinoma at pT1bN0M0 stage IA2 (Fig. 1b). Lung tumoroids were generated from the resected lung tumor as described above. The generated lung tumoroids (PDT-LUAD#5) were dense and irregular in shape while organoids derived from normal lung tissue (PDO-Normal#1, #16 and #36) appeared round and cystic (Fig. 1c and Supplementary Fig. 3a). Importantly, in the presence of nutlin-3a, lung tumoroids (PDT-LUAD#5) continued to grow while normal lung organoids (PDO-Normal#1, #16 and #36) ceased to grow (Fig. 1d right column). Karyotyping using fluorescence in situ hybridization (FISH) with an alpha-satellite probe indicated that normal lung organoids (PDO-Normal#1, #16 and #36) harbored a normal karyotype (2*n* = 46) whereas lung tumoroids (PDT-LUAD#5) harbored an aneuploid karyotype (2*n* = 64) (Fig. 1e and Supplementary Fig. 3b). Of note, a large chromosome indicated by an arrow has two alpha-satellite signals, suggesting the occurrence of a chromosomal rearrangement (Fig. 1e, left column enlarged image), which indicates that the lung tumoroids have chromosomal abnormalities that are not usually seen in normal lung epithelial cells (Fig. 1e, right column, Supplementary Fig. 3b). Since results by the certified laboratory did not detect any *EGFR* mutations or *ALK* fusions in the primary lung tumor, exome-seq was performed using genomic DNA extracted from the lung tumoroids to detect other possible therapeutically targetable driver oncogenes. Notably, a biallelic *TP53* mutation (c.463A > C, p.T155P) and a pathogenic *BRAF* mutation (c.1406G > C, p.G469A) were subsequently detected (Fig. 1f and Supplementary Table 2). Sanger sequencing confirmed these mutations in genomic DNA obtained from both the lung tumoroids and the parent lung tumor embedded in paraffin (Fig. 1g). Histopathological features of PDX inoculated from the lung tumoroids (PDT-LUAD#5) recapitulated those of the parent lung tumor (Fig. 1h). These results indicate that we successfully generated lung tumoroids that genetically and pathologically recapitulate the parent lung tumor, which can be used to develop a drug treatment strategy in case lung tumors recur. Since the lung tumoroids carry *BRAF*^*G469A*^ but not *BRAF*^*V600E*^, the Food and Drug Administration (FDA)-approved drug vemurafenib is not applicable for use in these patients. However, Yano and colleagues previously reported that the combination treatment of trametinib (a MEK inhibitor; FDA-approved for melanoma, colon cancer and NSCLC) and erlotinib (an EGFR inhibitor; FDA-approved for NSCLC) are effective to eradicate lung cancer cells that carry *BRAF*^*G469A*^ (Supplementary Fig. 4)^19^. Likewise, the combination treatment significantly suppressed the growth of the *BRAF*^*G469A*^ lung tumoroids (Fig. 1i, pink bar) and diminished ERK phosphorylation (a tumorigenic pathway marker) (Fig. 1j). These results indicate that lung tumoroids are useful to test different therapeutic strategies for each lung cancer patient.

### Generation of tumoroids derived from lymph node metastasis of metastatic lung adenocarcinoma

**Fig. 2:**
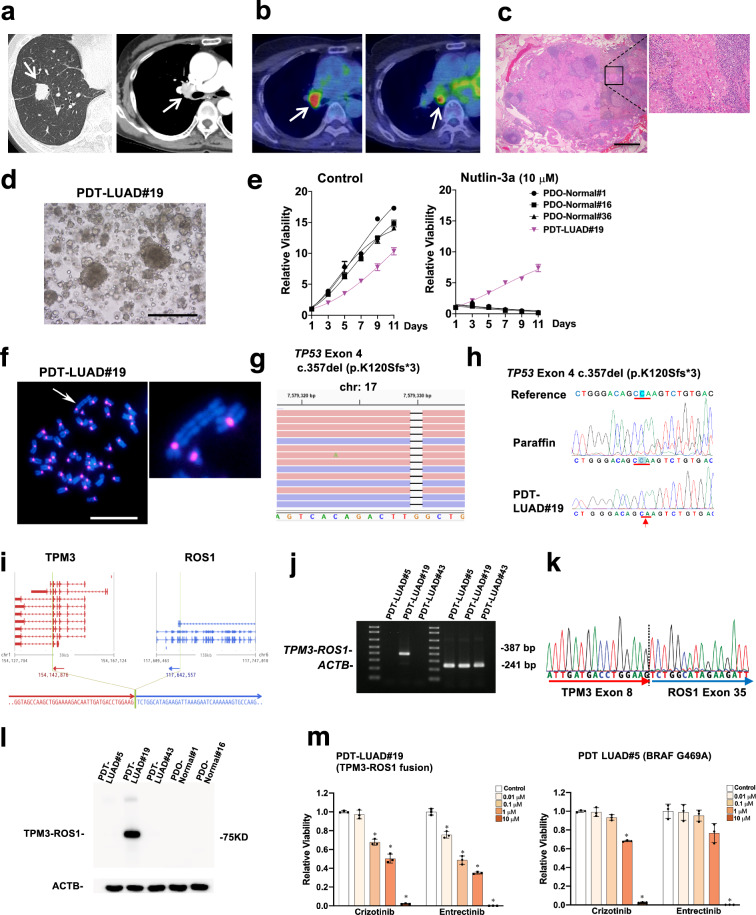
Patient-derived tumoroids (PDTs) from lymph node metastasis of a metastatic lung cancer (PDT-LUAD#19). **a** The nodule located in the right lower lobe was found by a CT scan (white arrow, left column). Enlarged right hilar lymph node was detected by Contrast Enhanced Computed Tomography (CECT) (white arrow, right column). **b** Increased FDG uptake in hilar (left) and subcarinal (right) lymph nodes was observed by FDG-PET/CT (white arrow). **c** Malignant cells were detected in the subcarinal lymph node specimen by HE staining (magnified on the right). Scale bar, 1 mm. **d** Shown is a bright-field microscopy image of PDT-LUAD#19. Scale bar, 500 μm. **e** Cell viability assays were conducted as described in Fig. 1d. **f** Metaphase FISH images are shown as described in Fig. 1e. An aberrant large chromosome was detected (white arrow). Scale bar, 20 μm. **g** Exome-seq detected c.357del (p.K120Sfs*3) and c.91G > A (p.V31I) (not shown) in both alleles of *TP53* from PDT-LUAD#19. Red and blue lines represent forward and reverse reads, respectively. **h** Sanger sequencing confirmed the exome-seq data shown in Fig. 2g. **i** RNA-seq detected a *TPM3-ROS1* fusion from PDT-LUAD#19. The junction of the two genes was located in chr1:154,142,876 and chr6:117,642,557. **j** RT-PCR confirmed the expression of a *TPM3-ROS1* transcript in PDT-LUAD#19. *ACTB* was used as internal control. **k** Sanger sequencing result of a RT-PCR product from PDT-LUAD#19 harboring a *TPM3-ROS1* rearrangement was consistent with the RNA-seq result. **l** TPM3-ROS1 fusion protein was detected using ROS1 antibody in PDT-LUAD#19. ACTB was used as internal control. **m** ROS1 inhibitors (crizotinib and entrectinib) significantly suppressed the growth of PDT-LUAD#19 (*TPM3-ROS1*; *TP53*^K120Sfs*3^) at lower concentration than that of PDT-LUAD#5 (*BRAF*^*G469A*^; *TP53*^*T155P*^) 72 h after treatment. Cell viability assays were performed as described in Fig. 1d. Data are shown as mean ± SD.

A 65-year-old woman was diagnosed with suspected lung cancer associated with a mediastinal lymph node metastasis using CT and positron emission tomography (PET) scans (Fig. 2a, b). Pathological examination from a trans-bronchial lung biopsy (TBLB) indicated lung adenocarcinoma. Right pneumonectomy and mediastinal lymphadenectomy were performed on the patient. Postsurgical pathological diagnosis confirmed lung adenocarcinoma at stage IIIA (Fig. 2c). Lung tumoroids (PDT-LUAD#19) were generated from a resected lymph node metastasis. Of note, we failed to generate lung tumoroids from a resected primary lung tumor. The PDT-LUAD#19 lung tumoroids were also dense and irregular in shape (Fig. 2d) as seen in the PDT-LUAD#5 lung tumoroids (Fig. 1c) and grew even in the presence of nutlin-3a while normal lung organoids did not (Fig. 2e), suggesting that the lung tumoroids harbor mutant p53. Karyotyping indicated that PDT-LUAD#19 lung tumoroids harbored an aneuploid karyotype (2*n* = 43) and an abnormally large chromosome, indicating that we succeeded in generating lung tumoroids (Fig. 2f). Since no *EGFR* mutations or *ALK* fusions were detected in the TBLB specimens according to the results by the certified laboratory, exome-seq was performed using genomic DNA extracted from the lung tumoroids. A biallelic *TP53* frame shift mutation (*TP53* c357del, p.K120Sfs*3) was detected (Fig. 2g, h and Supplementary Table 2); however, no therapeutically targetable driver mutations were detected. Thus, we also performed RNA-seq, which resulted in the identification of a *TPM3-ROS1* fusion in the lung tumoroids (Fig. 2i and Supplementary Table 2)^20^. Reverse transcription PCR (RT-PCR), Sanger sequencing, and immunoblots confirmed the *TPM3-ROS1* fusion in the lung tumoroids (Fig. 2j–l). FDA-approved drugs (crizotinib and entrectinib) for NSCLC that harbors *ROS1* fusions significantly suppressed the growth of the lung tumoroids (Fig. 2m)^21^, indicating that such FDA-approved drugs will be effective on the patient in case unresectable lung tumors recur in this patient. Of note, currently only one lung cell line (HCC78) carrying a *ROS1* fusion (*SLC34A2-ROS1*) is available from a public biobank (DSMZ-German Collection of Microorganisms and Cell Culture GmbH), thus this additional lung tumoroid line (*TPM3-ROS1*) that we generated here would be useful for the lung cancer research community.

### Generation of tumoroids derived from malignant pleural effusion of a lung cancer patient with acquired resistance to EGFR TKIs (tyrosine kinase inhibitors)

**Fig. 3:**
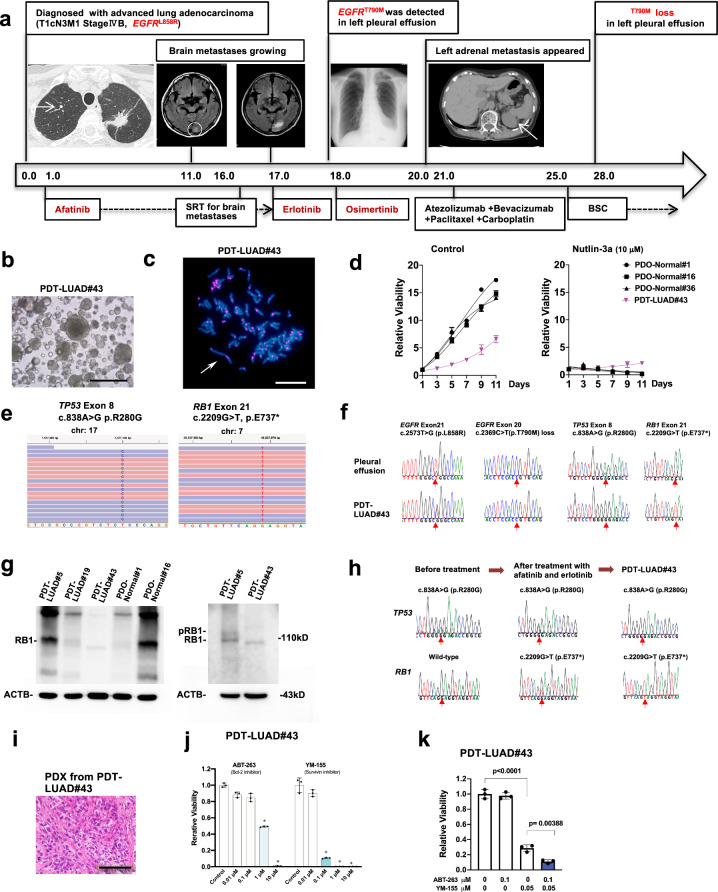
Patient-derived tumoroids (PDTs) from malignant effusion of lung cancer with acquired resistance to EGFR TKIs (tyrosine kinase inhibitors) (PDT-LUAD#43). **a** Timeline of treatment for a patient with *EGFR* mutations is shown. Numbers represent months since initial diagnosis. **b** Shown is a bright-field microscopy image of PDT-LUAD#43. Scale bar, 500 μm. **c** Metaphase FISH image is shown as described in Fig. 1e. Aberrant chromosome indicated by a white arrow was seen. Scale bar, 20 μm. **d** Cell viability assays were conducted as described in Fig. 1d. **e** Exome-seq detected c.838A > G (p.R280G) in both alleles of *TP53* and c.2209G > T (p.E737*) in both alleles of *RB1* from PDT- LUAD#43. Red and blue lines represent forward and reverse reads, respectively. **f** Sanger sequencing detected monoallelic *EGFR* c.2573T > G (p.L858R) alteration in addition to the exome-seq data shown in Fig. 3e. *EGFR* c.2369C > T (p.T790M) mutation was detected before osimertinib treatment but was lost in the effusion and the tumoroid. **g** Total RB1 was detected using RB1 antibody in all lung tumoroids (PDTs) and normal lung organoids (PDOs) except PDT-LUAD#43 (left panel). A phosphorylated RB1 band in PDT-LUAD#5 but not in PDT-LUAD#43 was observed using a longer running gel (right panel). **h** Sanger sequencing detected *TP53* c.838A > G (p.R280G) mutation in the biopsied specimen before any of the TKI treatment. *RB1* c.2209G > T (p.E737*) mutation was not detected before the TKI treatment, however the mutation appeared after afatinib and erlotinib treatment. **i** Shown is an HE stained image of PDX derived from PDT-LUAD#43. Scale bar, 100 μm. **j** Bcl-2 inhibitors (ABT-263) and a survivin inhibitor (YM-155) significantly suppressed the growth of PDT-LUAD#43 72 h after treatment. Cell viability assays were performed as described in Fig. 1d. Data are shown as mean ± SD. **k** Combination treatment of ABT-263 and YM-155 significantly suppressed the viability of PDT-LUAD#43 72 h after treatment. Data are shown as mean ± SD.

A 73-year-old woman was diagnosed with brain, skeletal, contralateral lung and lymph node metastases from a left lung adenocarcinoma at Stage IVB. The *EGFR* ^L858R^ was identified in her TBLB specimens from the primary tumor of the left upper lobe. Eighteen months after receiving treatment with afatinib and subsequent erlotinib (TKIs for the *EGFR*^*L858R*^), both the *EGFR*^*L858R*^ and the *EGFR*^T790M^ mutations were detected in the malignant pleural effusion, therefore her treatment regimen was switched to osimertinib (another TKI for mutant EGFR, including *EGFR*^*L858R*^ and *EGFR*^*T790M*^). Notably, 10 months after osimertinib treatment, the *EGFR*^T790M^ mutation was lost in the effusion; however, the *EGFR*^*L858R*^ was still detected. Lung tumoroids (PDT-LUAD#43) were generated from the effusion (Fig. 3a). The lung tumoroids were also dense and irregular in shape (Fig. 3b). Karyotyping identified an aneuploidy (2*n* = 82) and an aberrant large chromosome in the tumoroids, indicating that the tumoroids originated from the lung tumor cells, not normal lung epithelial cells (Fig. 3c). The lung tumoroids grew in the presence of nutlin-3a, suggesting that the tumoroids harbor mutant p53 (Fig. 3d). In order to identify the mechanism by which lung tumors became resistant to osimertinib, exome-seq was performed, resulting in the confirmation of *EGFR*^*L858R*^ and a p53 mutation (*TP53*^R280G^) and the identification of *RB1*^E737^* in both genomic alleles (Fig. 3e and Supplementary Table 2), the results of which were further confirmed by Sanger sequencing and immunoblots (Fig. 3f, g). *RB1* loss is one of the known mechanisms by which lung tumors become resistant to EGFR TKIs^22,23^. *RB1*^E737^* was detected after treatment with afatinib and erlotinib and retained even after treatment with osimertinib (Fig. 3h). Although co-mutation of p53 and *RB1* is frequently seen in small cell lung cancer (SCLC)^24^, PDX inoculated from the lung tumoroids displayed NSCLC but not SCLC (Fig. 3i). Since a Bcl-2 inhibitor (ABT-263; also known as navitoclax) and a survivin inhibitor (YM-155) have been reported to be effective to treat EGFR TKI-resistant lung cancers^22,25,26^, we treated the lung tumoroids with these inhibitors. Importantly, these inhibitors (single or in combination) significantly suppressed the growth of lung tumoroids (Fig. 3j, k). Although the treatment strategy using a Bcl-2 and/or survivin inhibitor has yet to be approved by the FDA, our current data using the lung tumoroids provide rationale for their use to treat this patient on a compassionate use basis.

### Which tumoroid culture system is the best?

**Fig. 4:**
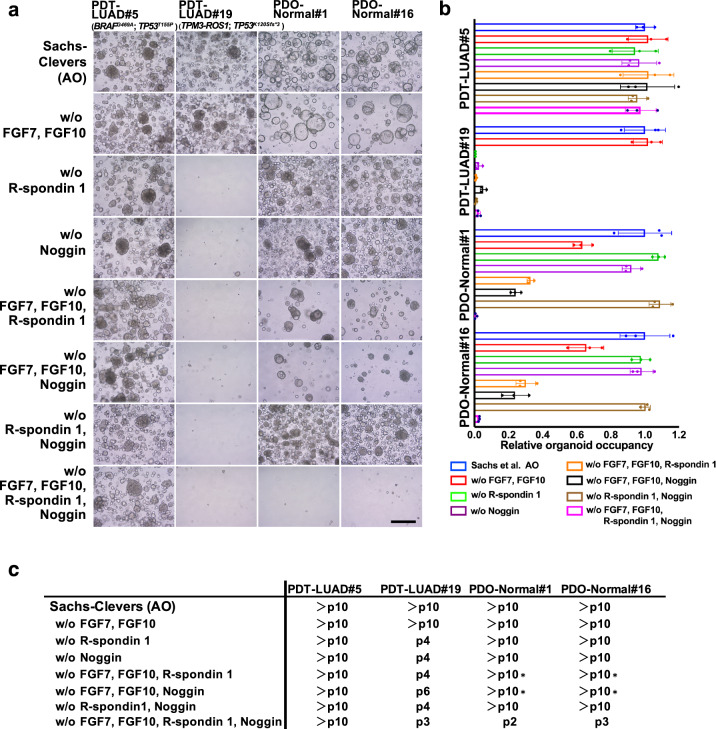
Effects on lung tumoroids (PDTs) and normal lung organoids (PDOs) by AO media modification. **a** Shown are bright-field microscopy images of PDTs and PDOs. Growth factors that comprise AO media were removed from AO media as indicated to determine which growth factors are indispensable for the growth of lung tumoroids and normal lung organoids. Representative images of the PDTs/PDOs in each condition at the passage number indicated in **c** are shown. Scale bar, 200 μm. **b** The growth of the tumoroids/organoids was quantified by measuring three independent areas of the tumoroid/organoid occupancy ratio in the images shown in **a**. Data are shown as mean ± SD. **c** Passage numbers of the PDTs and PDOs in the indicated media conditions are assessed to determine which condition are most suitable for culture. We noted that a number of the conditions slowed the growth of normal lung organoids though they were passaged over 10 times (depicted as *).

In this study, we used AO media for growing lung tumoroids^9^; however, since each generated tumoroid harbors a distinct driver mutation, we hypothesized that each of them might require different growth factors (but not all) in AO media and then sought to determine whether growth factors would be required for each lung tumoroid. As shown in Fig. 4, lung tumoroids from PDT-LUAD#5 (*BRAF*^*G469A*^; *TP53*^*T155P*^) grew long-term (>10 passages) in AO media lacking growth factors (FGF7, FGF10, R-spondin 1 and Noggin) while lung organoids from normal lungs of PDO-Normal#1 and PDO-Normal#16 did not grow (bottom panels). However, lung tumoroids from PDT-LUAD#19 (*TPM3-ROS1*; *TP53*^K120Sfs*3^) did not grow in AO media lacking the growth factors, indicating that modifying AO media may result in failure to generate a population of lung tumoroids.

**Fig. 5:**
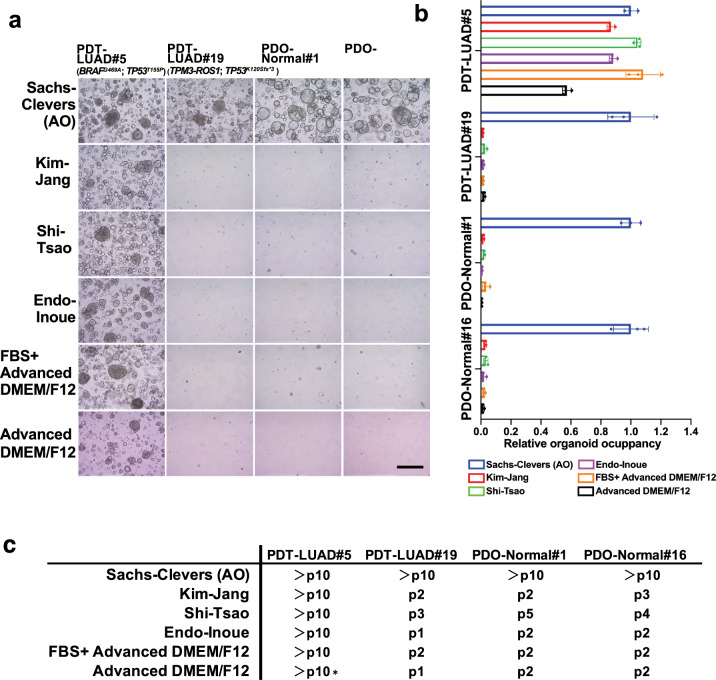
AO media is superior to other media on the growth of lung tumoroids (PDTs) and normal lung organoids (PDOs). **a** Shown are bright-field lignant effusion of lung cancer with acquired resincluding ones from three different laboratories (Kim-Jang, Shi-Tsao and Endo-Inoue) as indicated. Representative images of the PDTs/PDOs in each medium at the passage number indicated in **c** are shown. Scale bar, 200 μm. **b** The growth of the tumoroids/organoids was quantified as described in Fig. 4b. Data are shown as mean ± SD. **c** Passage numbers of the PDTs and PDOs in the indicated media are assessed to determine the best culturing conditions. We noted that advanced DMEM/F12 slowed the growth of PDT-LUAD#5 though it was passaged over ten times (depicted as *).

As mentioned in the Introduction, since Inoue, Jang, Tsao and colleagues (Endo-Inoue, Kim-Jang, Shi-Tsao, respectively) reported that they succeeded in generating long-term lung tumoroids at higher rates than we achieved using AO media^12,14,15^, we tested their media to determine whether their media would promote the growth of our lung tumoroids better than AO media (Supplementary Table 3). We also tested media using fetal bovine serum instead of specific growth factors (FBS only) and media without any serum or growth factors. Although other media were not particularly superior to AO media for the growth of lung tumoroids (PDT-LUAD#5; *BRAF*^*G469A*^; *TP53*^*T155P*^), they were sufficient for long-term culture of the lung tumoroids (>10 passages), indicating a population of lung tumoroids that grows only with minimum growth factors from Matrigel (Fig. 5). In contrast, PDT-LUAD#19 lung tumoroids (*TPM3-ROS1*; *TP53*^K120Sfs*3^) grew only in AO media but not in other media for the long term, indicating along with the data in Fig. 4 that R-spondin 1, SB 202190 and/or nicotinamide in the AO media are essential to culture PDT-LUAD#19 lung tumoroids, which suggests that tumorigenic pathways influenced by R-spondin 1, SB 202190 and/or nicotinamide might be possible therapeutic targets for ROS1 tyrosine kinase inhibitor-resistant lung cancers. The results were also replicated using the lung tumoroids before the first passage (Supplementary Fig. 5). These results suggest that AO media is superior to other media in order to culture different types of lung tumoroids for the long term.

## Discussion

Our major goal in this study was to generate avatar lung cancer cell lines from all of the lung cancer patients in our hospital using a recently developed lung tumoroid culture system for personalized medicine. However, the success rate for generating lung tumoroids was only 7% (3/41). These results were consistent with the results reported by Swanton, Voest and colleagues (8% and 17%, respectively)^16,17^. The major reason for this low success rate was because the current tumoroid culture systems allow lung organoids from normal lung epithelial cells to grow faster than lung tumoroids. The results were surprising to us since our study group includes a thoracic surgeon (T. Fukazawa) and a pathologist (Y. Monobe) specializing in lung cancer, both of whom carefully collected lung tumor specimens together for this study from resected lung tumor tissues to avoid contamination of surrounding normal lung tissues. In order to eliminate such normal lung organoids, we used nutlin-3a, thereby failing to generate lung tumoroids that harbor wild-type p53. Since ~40% of lung cancers harbor wild-type p53 but not mutant p53 according to TCGA^24,27,28^, it is essential to develop a lung tumoroid culture system in which nutlin-3a is not required. Recently, Sato and colleagues succeeded in culturing RB1-mutant gastroenteropancreatic neuroendocrine neoplasms using palbociclib, a CDK4 inhibitor^29^. Palbociclib may be useful to generate RB1-mutant and wild-type p53 lung tumoroids. We also tested other lung tumoroid culture media reported by Inoue, Jang, Tsao and colleagues;^12,14,15^ however, those media failed to culture one of our lung tumoroid lines (PDT-LUAD#19 harboring *TPM3-ROS1*; *TP53*^K120Sfs*3^) for the long term, suggesting that AO media with nutlin-3a is superior to them. The media reported by Inoue, Jang, Tsao and colleagues may be suitable for culturing lung tumoroids derived from lung cancers that harbor wild-type p53. In the present study, we cultured all of the freshly dissociated lung tumor cells in the AO media. However, for future studies, we plan to culture such freshly dissociated lung tumor cells in different media separately along with the AO media, which may expand the feasibility to generate more genetically and/or pathologically different types of lung tumoroids.

Although the success rate for generating lung tumoroids was low, none of our lung tumoroids grew in the 2D cell culture system that lacks Matrigel (data not shown), indicating that the lung tumoroid culture system enables us to culture a population of lung cancer cells, which were not previously possible to culture using the 2D culture system. If the current lung tumoroid system is combined with the 2D culture system, we will be able to generate avatar lung cancer cell lines at a success rate of 10–20%, which may still be useful for personalized medicine.

Of note, among the three lung tumoroid lines that we generated in this study, one out of 28 primary lung cancers, one out of 1 lymph node metastasis and one out of 2 malignant pleural effusions were generated, suggesting that the success rate may increase if lung tumoroids are generated only from metastatic (but not primary) lung cancers. Likewise, the success rate of generating lung cancer cell lines using the 2D culture system from metastatic lung cancers is higher than that from primary lung cancers^5^. Generating avatar lung tumoroids from patients with lymph node metastases or malignant pleural effusion is valuable since such metastatic lung cancers often recur or progress even after surgical resection and/or first-line drug treatment. Using the avatar lung tumoroids, clinicians can test the efficacy of first-line and next-line drugs, drugs in clinical trials and/or experimental drugs before the treatment of patients in the clinic. Although the success rate of generating lung tumoroids has to be improved, our present study indicates that a lung tumoroid culture system is useful for designing a therapeutic strategy for each lung cancer patient.

## Methods

### Patient-derived tumoroid (cancer organoid) culture (PDT) system

Human lung PDTs were generated using a tumoroid culture system previously reported by the laboratories of Clevers^11^, Jang^14^, Tsao^15^ and Inoue^12^. The detailed system developed by Clevers and colleagues^9^ is available from PMCID: PMC6376275. The detailed media components used by each laboratory are described in Supplementary Table 3. For lung tumor detection, a computer tomography (CT) and/or positron emission tomography (PET) scans were conducted using Aquilion PRIME TSX-303A (Canon Medical Systems, Tochigi, Japan) and Discovery IQ (GE Healthcare Japan, Tokyo, Japan). For video-assisted thoracic surgery to resect lung tumor, thoracoscope (WA50373B, Olympus Medical Systems Corp, Tokyo, Japan) was used. The research protocol was approved by the Ethics Committee of Kawasaki Medical School (Reference Number: 3171-1). All patients participating in this study signed informed consent forms that were approved by the responsible authority.

### RT-PCR-mediated detection of *TPM3-ROS1*

Total cellular RNA was extracted from cells using TRIzol (Thermo Fisher Scientific). Reverse transcription (RT) was performed using PrimeScript™ RT reagent Kit (Takara Bio, Shiga, Japan). PCR was performed using TaKaRa Ex Taq (Takara Bio). The primers used for the amplification of *TPM3-ROS1* and *ACTB* are described in Supplementary Table 4.

### Luminescent viability assay

Viability was determined using the CellTiter-Glo^R^ 2.0 Cell Viability Assay (Promega, Madison, WI) according to the manufacturer’s instructions. Luminescence was measured using a Varioskan LUX Multimode Microplate Reader (Thermo Fisher Scientific).

### Fluorescence in situ hybridization (FISH)

The alpha-satellite DNA for all chromosomes was generated by PCR from human genomic DNA (Takara Bio) using PCR primers 5′-GAAGCTTA(A/T)(C/G)T(C/A)ACAGAGTT(G/T)AA-3′ and 5′-GCTGCAGATC(A/C)C(A/C)AAG(A/T/C)AGTTTC-3′^30^ labeled by Nick Translation Mix (Sigma-Aldrich) with rhodamine (magenta). FISH was performed using a standard protocol^31^. Slides were counterstained with DAPI and analyzed with a fluorescence microscope (Olympus BX53, Olympus, Japan).

### Immunoblot analysis and immunohistochemistry

Immunoblot analysis and immunohistochemistry were performed as previously described^32^. Antibodies used in the study are shown in Supplementary Table 5. All blots or gels derive from the same experiment and they were processed in parallel. Unprocessed images are shown in Supplementary Figs. (see page 7–9).

### Sanger sequencing and next-generation sequencing

Genomic DNA was isolated from tumoroids/organoids using QIAamp DNA Mini Kit (Qiagen, Hilden, Germany) or from paraffin tissue using DEXPAT (Takara Bio) according to manufacturer’s protocol. PCR reactions were performed using TaKaRa Ex Taq (Takara Bio) with the primers (Supplementary Table 4). Sanger sequencing was performed by Eurofins Genomics K. K. (Tokyo, Japan).

### Next-generation sequencing

Next-generation sequencing, including exome-seq and RNA-seq, was performed by Riken Genesis (Tokyo, Japan). For exome-seq, cutadapt (v1.2.1) and Picard (ver.1.73) were used to remove adapter sequences and alignment duplicates. BWA (ver.0.7.10) was used for read alignment to hg19 reference genome. GATK (ver.1.6–13) was used for SNV and Indel calling. For RNA-seq, cutadapt (v1.2.1) and PRINSEQ (v0.19.2) were used for quality control, removing adapter, low-quality and poly-A/T sequences. And deFuse (v0.6.2) was used to detect potential gene fusions.

### PDX inoculated from lung tumoroids

Lung tumoroids were dissociated using TrypLE (Thermo Fisher Scientific). 1.2 × 10^6^ cells from lung tumoroid PDT-LUAD#5 or 1.5 × 10^6^ cells from lung tumoroid PDT-LUAD#43, which were mixed with 50 μl of BME2 (Matrigel), were subcutaneously injected into the 5-week-old dorsal flank of NOD/SCID female mice (Charles River Laboratories Japan, Atsugi, Japan). The experimental protocol was approved by the Ethics Review Committee for Animal Experimentation of Kawasaki Medical School (Ethics Committee reference number: 19-039-1).

### Statistical analysis

Results are expressed as the mean ± SD of the replicates for each group. Differences between two study groups were evaluated using Student’s *t*-test (two-sided). *P*-value < 0.01 was considered significant.

### Reporting summary

Further information on research design is available in the Nature Research Reporting Summary linked to this article.

## Supplementary information

REPORTING SUMMARY

Supplementary Information

Supplementary Table 1-5

## Data Availability

The data generated and analyzed during this study are described in the following data record: 10.6084/m9.figshare.13802894^33^. The RNA-seq data are openly available in the Gene Expression Omnibus repository under accession https://identifiers.org/geo:GSE157659^34^, and the exome-seq data are openly available in the Sequence Read Archive repository under accession code https://identifiers.org/ncbi/bioproject:PRJNA689916^35^. All other relevant data are available from the authors except for the information to identify participating patients.
