# Supplementary material for: Clinical application of a lung cancer organoid (tumoroid) culture system
Source: NPJ Precis Oncol. 2021 Apr 12;5:29. doi: 10.1038/s41698-021-00166-3 (PMC8042017; doi:10.1038/s41698-021-00166-3)
Supplement: Supplementary file 1 — REPORTING SUMMARY [file 41698_2021_166_MOESM1_ESM.pdf]

nature research

Corresponding author(s): Takuya Fukazawa

Last updated by author(s): Feb 14, 2021

Reporting Summary

Nature Research wishes to improve the reproducibility of the work that we publish. This form provides structure for consistency and transparency in reporting. For further information on Nature Research policies, see our Editorial Policies and the Editorial Policy Checklist.

Statistics

For all statistical analyses, confirm that the following items are present in the figure legend, table legend, main text, or Methods section.

n/a	Confirmed	
☐	☒	The exact sample size (n) for each experimental group/condition, given as a discrete number and unit of measurement
☐	☒	A statement on whether measurements were taken from distinct samples or whether the same sample was measured repeatedly
☐	☒	The statistical test(s) used AND whether they are one- or two-sided<br>*Only common tests should be described solely by name; describe more complex techniques in the Methods section.*
☒	☐	A description of all covariates tested
☒	☐	A description of any assumptions or corrections, such as tests of normality and adjustment for multiple comparisons
☐	☒	A full description of the statistical parameters including central tendency (e.g. means) or other basic estimates (e.g. regression coefficient) AND variation (e.g. standard deviation) or associated estimates of uncertainty (e.g. confidence intervals)
☒	☐	For null hypothesis testing, the test statistic (e.g. F, t, r) with confidence intervals, effect sizes, degrees of freedom and P value noted<br>*Give P values as exact values whenever suitable.*
☒	☐	For Bayesian analysis, information on the choice of priors and Markov chain Monte Carlo settings
☒	☐	For hierarchical and complex designs, identification of the appropriate level for tests and full reporting of outcomes
☒	☐	Estimates of effect sizes (e.g. Cohen's d, Pearson's r), indicating how they were calculated

Our web collection on statistics for biologists contains articles on many of the points above.

Software and code

Policy information about availability of computer code

Data collection	RNA-seq and exome-seq data were collected using next-generation sequencing, which are documented in the METHODS and public repository sites (Gene Expression Omnibus and Sequence Read Archive) under accession codes (GSE157659 and PRJNA689916, respectively).
Data analysis	The data collected using next-generation sequencing were analyzed as described in the METHODS. GraphPad Prism 8 was used for display and statistical analysis of cell viability and relative occupancy.

For manuscripts utilizing custom algorithms or software that are central to the research but not yet described in published literature, software must be made available to editors and reviewers. We strongly encourage code deposition in a community repository (e.g. GitHub). See the Nature Research guidelines for submitting code & software for further information.

Data

Policy information about availability of data

All manuscripts must include a data availability statement. This statement should provide the following information, where applicable:

- Accession codes, unique identifiers, or web links for publicly available datasets
- A list of figures that have associated raw data
- A description of any restrictions on data availability

Data availability statement is included in the manuscript.

Field-specific reporting

Please select the one below that is the best fit for your research. If you are not sure, read the appropriate sections before making your selection.

☒ Life sciences ☐ Behavioural & social sciences ☐ Ecological, evolutionary & environmental sciences

For a reference copy of the document with all sections, see nature.com/documents/nr-reporting-summary-flat.pdf

Life sciences study design

All studies must disclose on these points even when the disclosure is negative.

Sample size	Three cases of lung tumoroids were analyzed from 41 cases of lung cancer since we succeeded in generating lung tumoroids only from these three cases.
Data exclusions	We excluded 38 cases of lung cancer from our analysis since we failed to generate tumoroids from these cases.
Replication	Next-generation data were replicated by Sanger sequencing, RT-PCR and/or immunoblot analyses.
Randomization	This is not relevant to our study since we analyzed each lung tumoroid case based on its distinct genetic results.
Blinding	Next-generation sequencing was performed by Riken Genesis, which belongs to a different entity from Kawasaki Medical School. Patient informations was not provided to Riken Genesis prior to next-generation sequencing.

Reporting for specific materials, systems and methods

We require information from authors about some types of materials, experimental systems and methods used in many studies. Here, indicate whether each material, system or method listed is relevant to your study. If you are not sure if a list item applies to your research, read the appropriate section before selecting a response.

Materials & experimental systems

n/a	Involved in the study	
☐	☒	Antibodies
☐	☒	Eukaryotic cell lines
☒	☐	Palaeontology and archaeology
☐	☒	Animals and other organisms
☐	☒	Human research participants
☒	☐	Clinical data
☒	☐	Dual use research of concern

Methods

n/a	Involved in the study	
☒	☐	ChIP-seq
☒	☐	Flow cytometry
☒	☐	MRI-based neuroimaging

Antibodies

Antibodies used	All antibodies used in this study are described in Supplementary Table 5.
Validation	All of the antibody manufacturer's names are also described in Supplementary Table 5. The validation information is available from their websites.

Eukaryotic cell lines

Policy information about cell lines

Cell line source(s)	Human lung adenocarcinoma cell lines H1395 and H1666 used in this study were newly obtained from ATCC.
Authentication	Authentication was performed by ATCC.
Mycoplasma contamination	ATCC certifies that the cell lines are not contaminated with Mycoplasma.
Commonly misidentified lines (See ICLAC register)	H1395 and H1666 are not commonly misidentified cell lines.

Animals and other organisms

Policy information about studies involving animals; ARRIVE guidelines recommended for reporting animal research

Laboratory animals	The information is documented in the METHODS.
Wild animals	This study did not involve wild animals.
Field-collected samples	This study did not involve samples collected from the field.
Ethics oversight	The study protocol was approved by the Ethics Review Committee for Animal Experimentation of Kawasaki Medical School (Ethics Committee reference number: 19-039-1).

Note that full information on the approval of the study protocol must also be provided in the manuscript.

Human research participants

Policy information about studies involving human research participants

Population characteristics	Population characteristics is described in Table 1.
Recruitment	Participants were recruited from lung cancer patients at Kawasaki Medical School after signing informed consent forms. We do not see any potential biases that may impact results.
Ethics oversight	The study protocol was approved by the Ethics Committee of the Kawasaki Medical School (Ethics Committee Reference Number: 3171-1).

Note that full information on the approval of the study protocol must also be provided in the manuscript.
