# Supplementary material for: Clinical application of a lung cancer organoid (tumoroid) culture system
Source: NPJ Precis Oncol. 2021 Apr 12;5:29. doi: 10.1038/s41698-021-00166-3 (PMC8042017; doi:10.1038/s41698-021-00166-3)

SUPPLEMENTARY FIGURES

Clinical Application of a Lung Cancer Organoid (Tumoroid) Culture System

Etsuko Yokota, Miki Iwai, Takuro Yukawa, Masakazu Yoshida, Yoshio Naomoto,

Minoru Haisa, Yasumasa Monobe, Nagio Takigawa, Minzhe Guo, Yutaka Maeda,

Takuya Fukazawa and Tomoki Yamatsuji

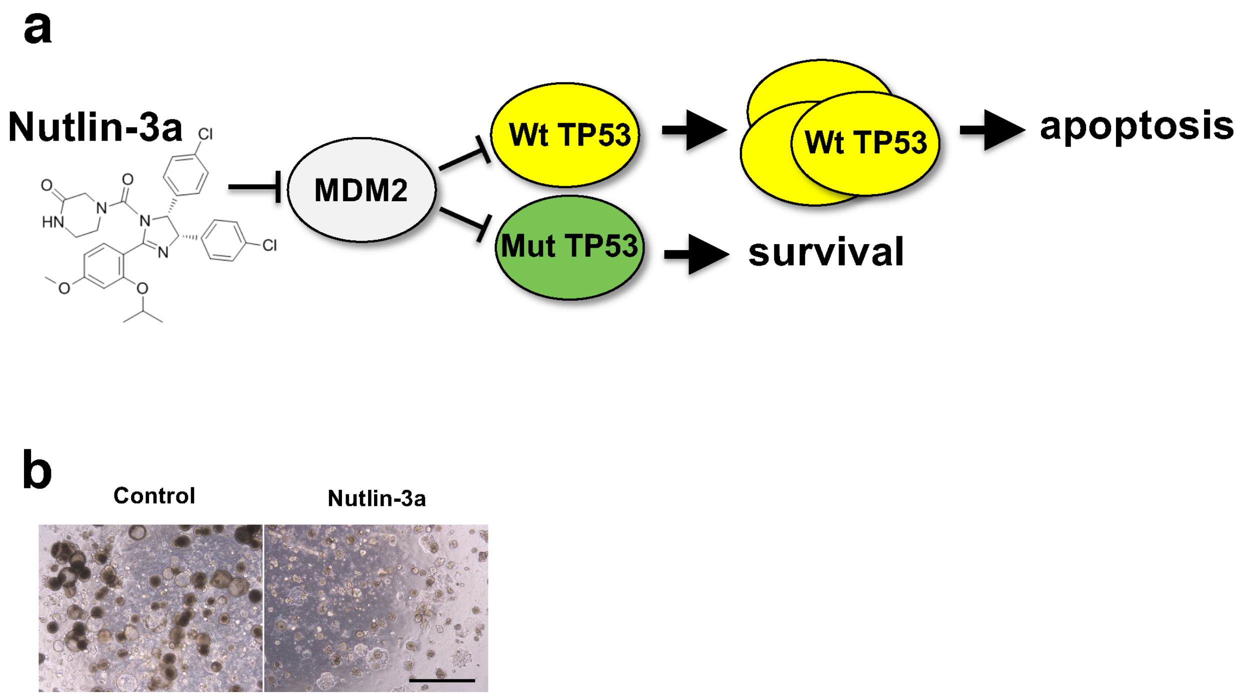

Supplementary Figure 1. Nutlin-3a inhibited the growth of normal lung organoids derived from normal lung tissue.

(a) Schematic model for wild-type (Wt) TP53 activation by nutlin-3a. Nutlin-3a inhibits the TP53-MDM2 interaction, thereby activating the TP53 pathway, which results in apoptosis of the cells, including normal lung epithelial cells, that harbor wild-type p53 but not mutant (Mut) p53.

(b) Nutlin-3a (10 μM) eliminated normal lung organoids derived from normal lung epithelial cells 9 days after treatment. Scale bar, 1 mm.

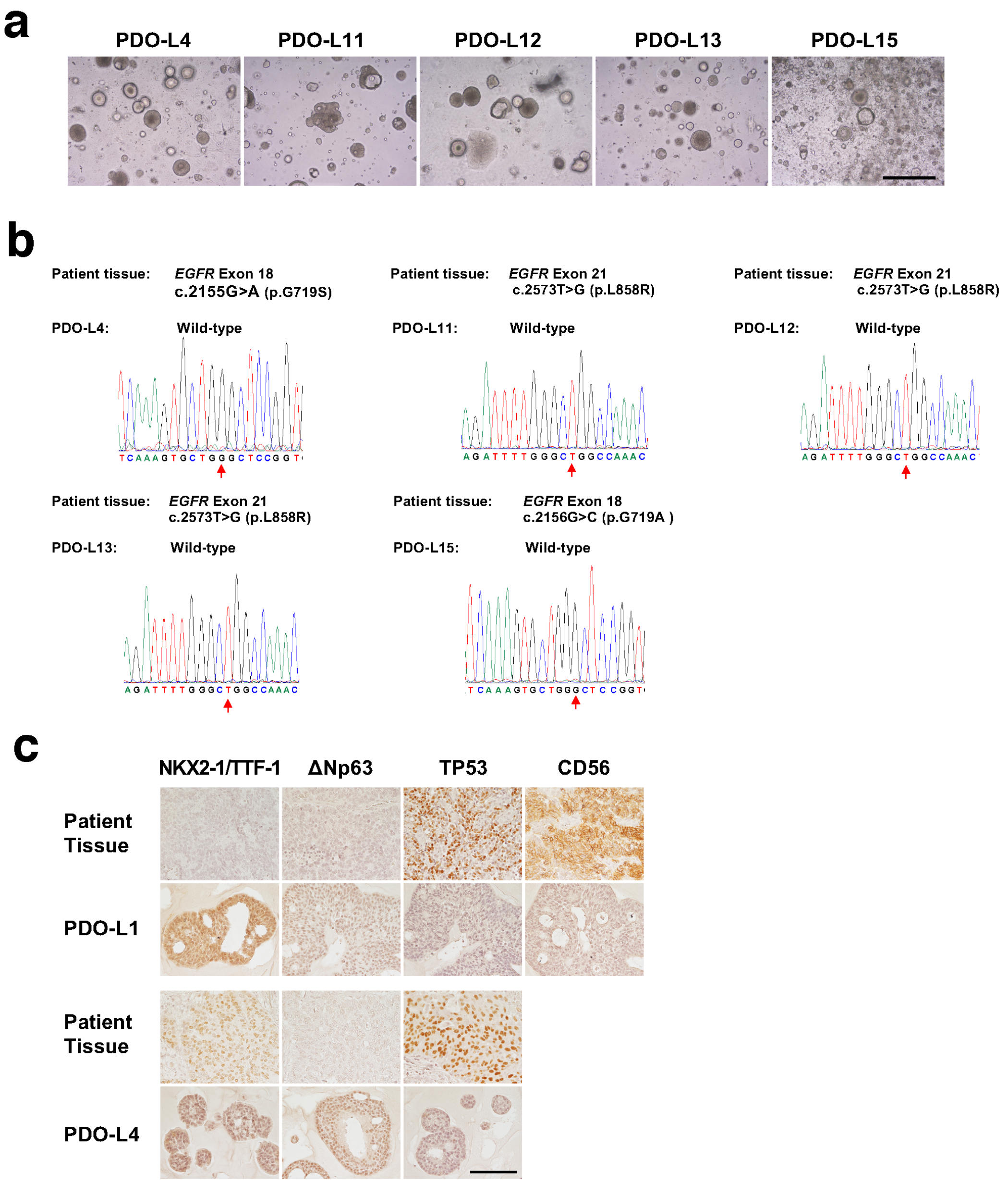

Supplementary Figure 2. Only normal lung organoids (PDOs) but not lung tumoroids (PDTs) were grown in AO media without nutlin-3a.
(a) Shown are bright-field microscopy images of PDOs (not PDTs; see b and c below) cultured without nutlin-3a for a week. All these 5 PDOs were derived from tissues of lung tumors that harbor *EGFR* mutations, details of which are shown in Table 1. Scale bar, 500 μm.
(b) Sanger sequencing using genomic DNA extracted from PDOs shown in (a) indicated that these PDOs did not harbor *EGFR* mutations. Hence, these PDOs were not PDTs though they are from lung tumor tissues.
(c) Shown are immunohistochemistry (IHC) images of formalin-fixed & paraffin-embedded patient lung tumors and PDOs derived from the lung tumor tissues. PDOs were first embedded in iPGel (NIPPON Genetics) and fixed with 4% paraformaldehyde overnight, then embedded in paraffin and cut in 4 μm thick sections. The expression of lung cancer markers in the patient lung tumors is not consistent with that in the PDOs. Scale bar, 100 μm.

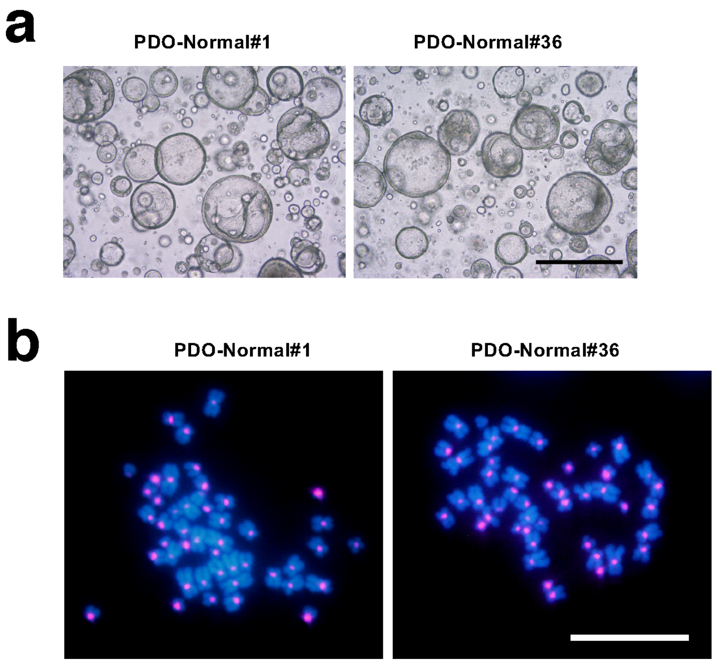

Supplementary Figure 3. Normal lung organoids (PDOs) look cystic and display normal karyotype.
(a) Shown are bright-field microscopy images of PDO-Normal#1 and PDO-Normal#36. These organoids were generated from peripheral normal lung tissue. Scale bar, 500 μm.
(b) Shown are metaphase FISH images of PDO-Normal#1 and PDO-Normal#36 using an alpha-satellite probe labeled with rhodamine (magenta), which display normal diploid karyotypes ($2n$=46). Scale bar, 20 μm.

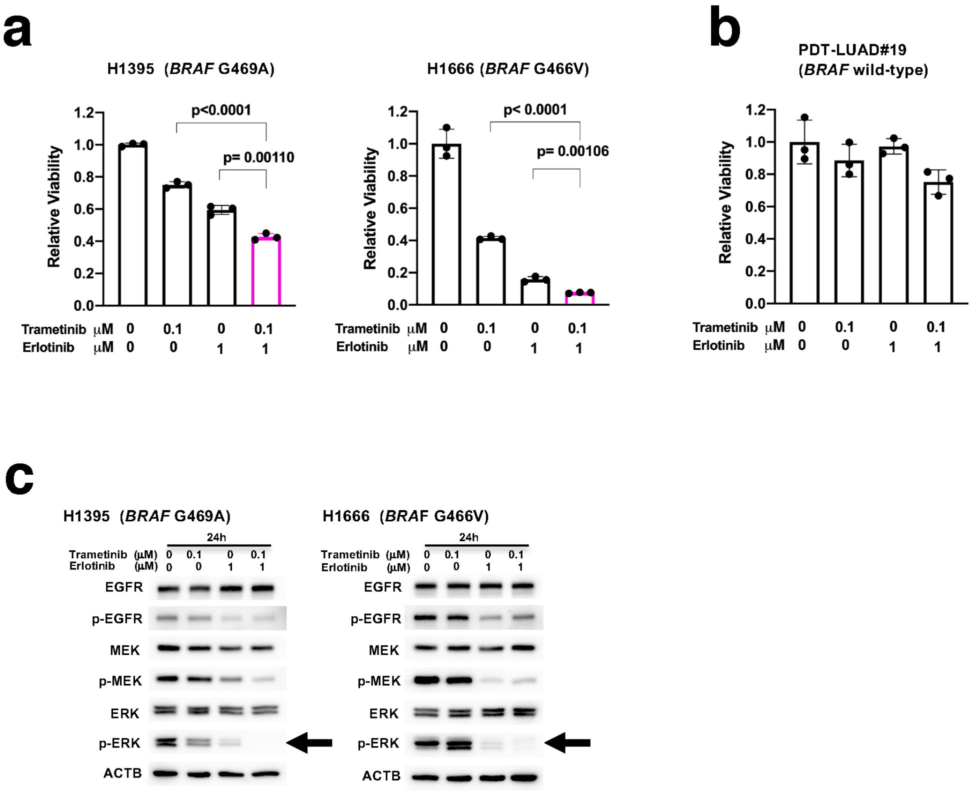

Supplementary Figure 4. Combination treatment of trametinib with erlotinib significantly suppressed cell viability of lung adenocarcinoma cells with non-V600E *BRAF* mutations.
(a) H1395 cells harboring *BRAF*^{G469A} and H1666 cells harboring *BRAF*^{G466V} that were obtained from ATCC (Manassas, VA) and grown in RPMI 1640 supplemented with 10% heat-inactivated fetal bovine serum (FBS). H1395 and H1666 cells were seeded into 96-well white polystyrene plates at a density of 1 x 10^3 cells per well 24 hours before treatment. Cell viability assays were performed as described in Materials and Methods section after treated with the indicated drugs for 72 hours. Data are shown as mean ± SD.
(b) Trametinib and/or erlotinib did not influence the cell viability of lung tumoroids that harbor wild-type *BRAF* 72 hours after treatment. Cell viability assay was performed as described in Fig. 1d. Data are shown as mean ± SD.
(c) Combination of trametinib with erlotinib suppressed the phosphorylation of ERK (arrows) in H1395 and H1666 cells that harbor non-V600E *BRAF* mutations 24 hours after treatment. Immunoblotting was performed as described in Materials and Methods section.

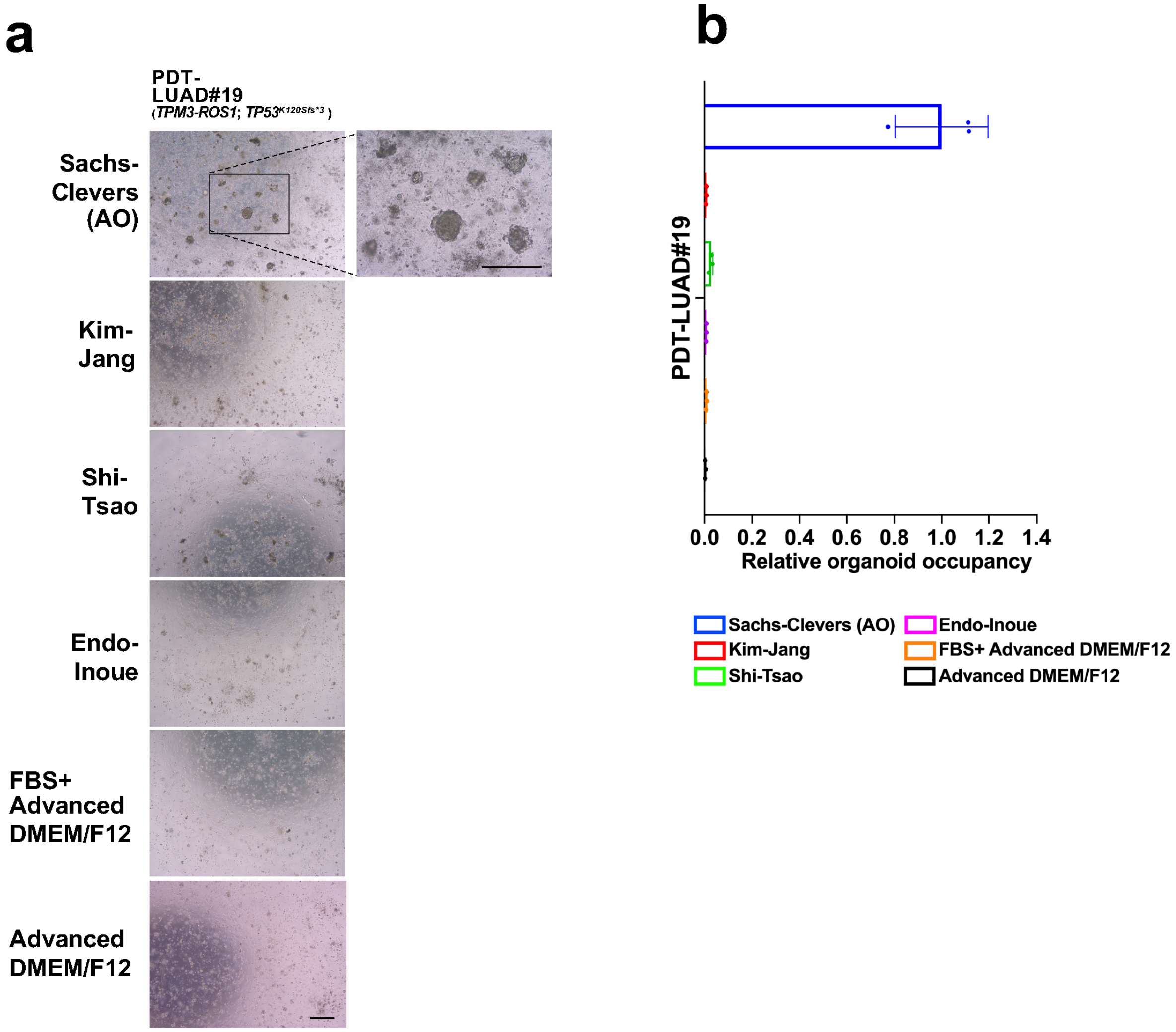

Supplementary Figure 5. Superiority of AO media compared to other media on the growth of lung tumoroids (PDTs) was replicated using PDT-LUAD#19 at the first passage from a frozen stock.

(a) Shown are representative bright-field microscopy images of PDTs grown in AO media and other media, including ones from 3 different laboratories (Kim-Jang, Shi-Tsao and Endo-Inoue) as indicated. Representative images of the PDTs in each medium cultured for 23 days after PDT-LUAD#19 (*TPM3-ROS1*; *TP53*^{K120Sfs*3}) was thawed from a frozen stock prepared before the first passage. Scale bar, 500 μm.

(b) The growth of the tumoroids was quantified as described in Fig. 4b. Data are shown as mean ± SD.

Figure 1j

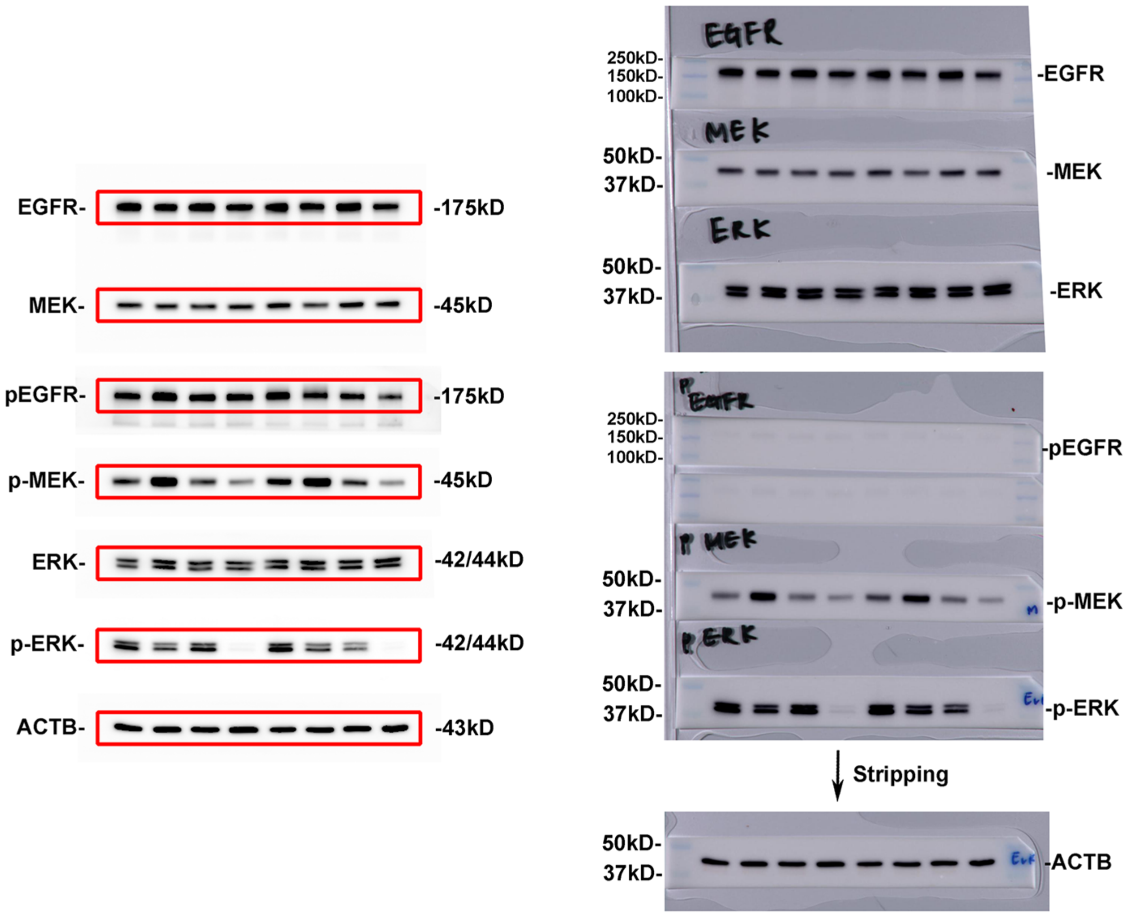
EGFR-
-175kD
MEK-
-45kD
pEGFR-
-175kD
p-MEK-
-45kD
ERK-
-42/44kD
p-ERK-
-42/44kD
ACTB-
-43kD
EGFR
250kD-
150kD-
100kD-
-EGFR
MEK
50kD-
37kD-
-MEK
ERK
50kD-
37kD-
-ERK
250kD-
150kD-
100kD-
-pEGFR
50kD-
37kD-
-p-MEK
50kD-
37kD-
-p-ERK
Stripping
50kD-
37kD-
-ACTB

Figure 2l

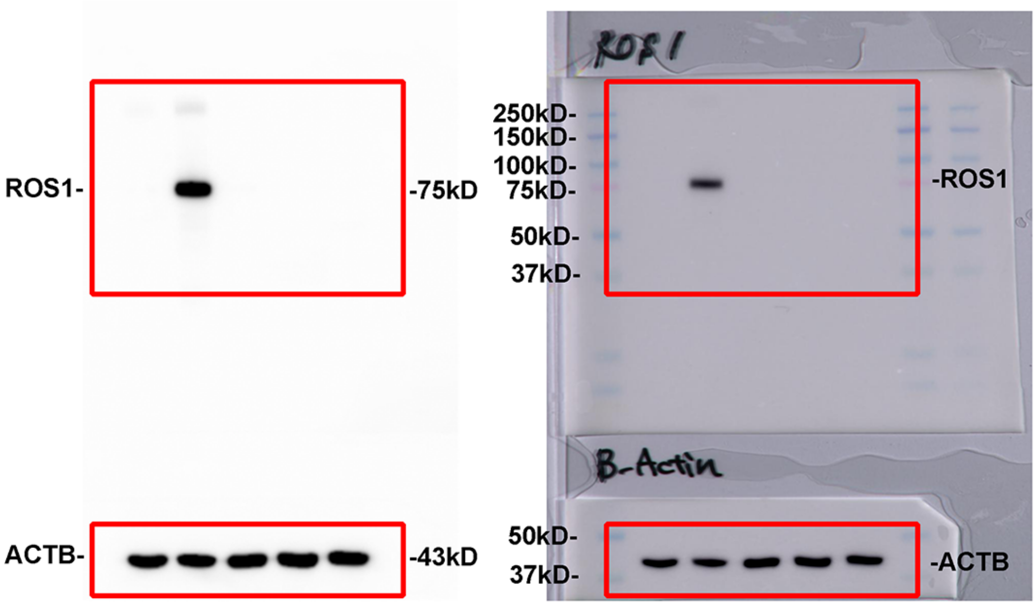

Figure 3g

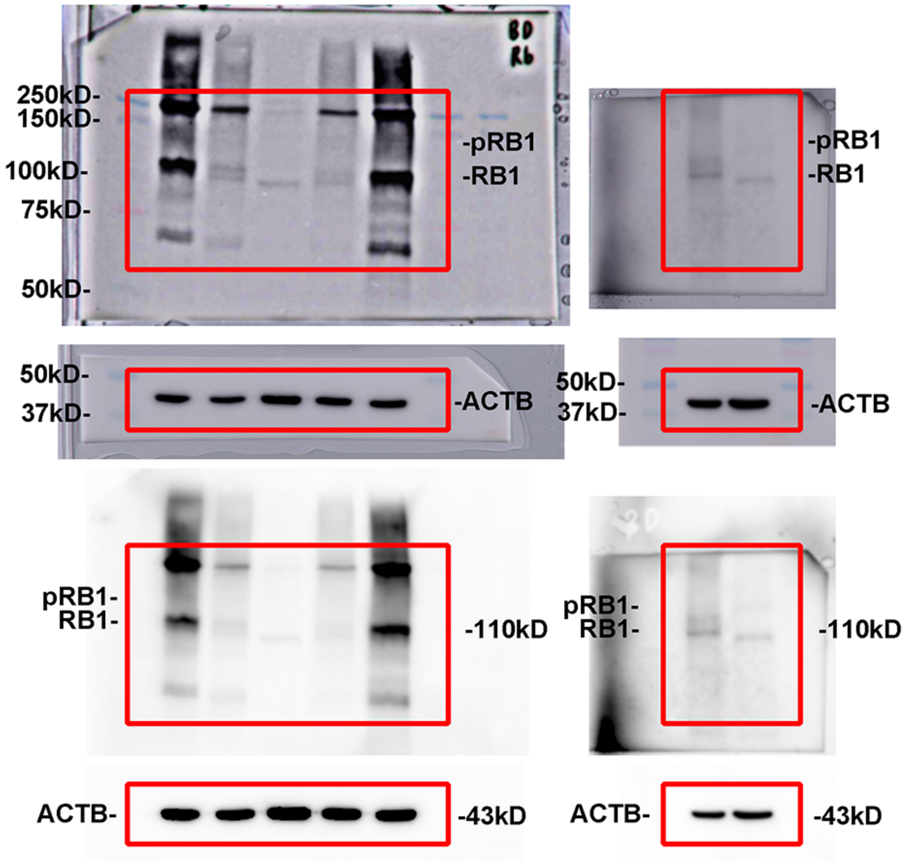

Supplementary Figure 4c

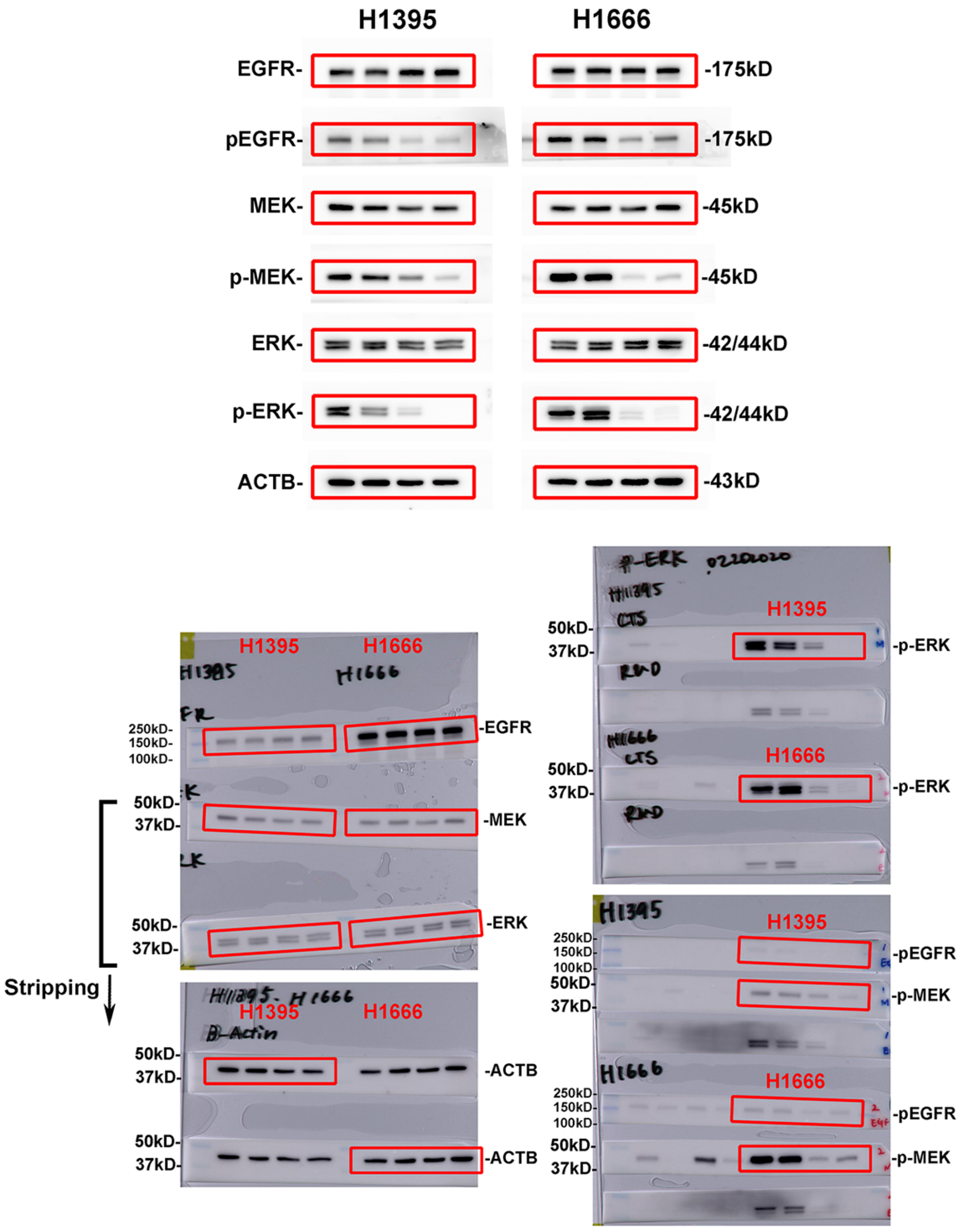

Supplement: Supplementary file 2 — Supplementary Information [file 41698_2021_166_MOESM2_ESM.pdf]
